# Advanced Insights Into Intravascular Bloodstream Infections: A Cross-Sectional Study of the Molecular Characterization of World Health Organization Priority Pathogens and Antimicrobial Resistance Patterns at a Tertiary Care Hospital

**DOI:** 10.7759/cureus.93375

**Published:** 2025-09-27

**Authors:** Likhitha Reddy A, Ramani CP, Sudha N, Saravana Priya JK

**Affiliations:** 1 Medicine, Madras Medical College, Rajiv Gandhi Government General Hospital, Chennai, IND; 2 Institute of Microbiology, Madras Medical College, Rajiv Gandhi Government General Hospital, Chennai, IND

**Keywords:** antibiotic stewardship program, bloodstream infections, carbapenemase genes, catheter-related infections, clinical outcomes, esbl producers, infective endocarditis, molecular resistance markers, multidrug-resistant pathogens, pcr diagnostics

## Abstract

Introduction

Bloodstream infections (BSIs), particularly intravascular types such as catheter-related bloodstream infections (CRBSIs) and infective endocarditis (IE), represent a significant public health challenge due to their high morbidity and potential to rapidly progress to sepsis. The emergence of multidrug-resistant (MDR) pathogens necessitates molecular surveillance to inform effective antimicrobial strategies, especially in regions like India, where resistance data is limited.

Methods

A cross-sectional study was conducted over seven months, enrolling 101 patients from cardiology, nephrology, and ICUs at a tertiary care hospital in Chennai. Patients with confirmed intravascular BSIs were identified using clinical criteria and modified Duke’s definitions. Blood cultures and catheter tip analyses were performed using standard microbiological techniques. Phenotypic resistance mechanisms were assessed via extended-spectrum beta-lactamase (ESBL), methicillin resistance, and CARBA NP testing. Molecular characterization included polymerase chain reaction (PCR) detection of resistance genes (blaKPC, blaNDM, blaCTX-M, blaTEM, OXA-23, OXA-48, mecA). Data were analyzed using IBM SPSS Statistics Version 31.0 (IBM Corp., Armonk, NY). Means and proportions were reported with 95% confidence intervals (CIs).

Results

The cohort included patients aged 13-68 years, with a male predominance (70 patients, 70%). CRBSIs accounted for 86 cases (85%). All patients underwent internal jugular vein (IJV) catheterization, primarily for hemodialysis in chronic kidney disease (CKD) (68 patients, 68%). Blood culture positivity was observed in 49 patients (48.5%), with Klebsiella pneumoniae (14 patients, 13.9%), Pseudomonas spp. (10 patients, 9.9%), and Staphylococcus aureus (8 patients, 7.9%) as dominant isolates. ESBL production was highest in Escherichia coli (four out of five isolates, 80%) and Klebsiella (10 out of 14 isolates, 71.4%). Carbapenemase genes blaNDM and blaKPC were detected in five out of 14 Klebsiella isolates (35.7%), while OXA-23 was found in five out of six Acinetobacter isolates (83.3%). A significant correlation was found between catheter duration and culture positivity using a scatter plot demonstration. Targeted antimicrobial therapy was successful in 71 patients (70%), although complications such as persistent bacteremia and septic shock were noted.

Conclusions

This study underscores the high prevalence of CRBSIs and the concerning presence of molecular resistance markers in key pathogens. The predominance of carbapenemase genes such as blaNDM, blaKPC, and OXA variants highlights the urgent need for molecular diagnostics and robust antibiotic stewardship in India. Continued surveillance of intravascular BSIs and resistance profiles is essential to improve clinical outcomes and combat antimicrobial resistance.

## Introduction

Bloodstream infections (BSIs) can progress to life-threatening sepsis, contributing to high morbidity and mortality worldwide. Studies indicate that annual BSI episodes in Europe range from 1,213,460 to 1,381,590, accompanied by substantial BSI-associated mortality [1]. However, data on BSI profiles from nations like India remain limited [2]. Intravascular infections, originating within the cardiovascular system, pose severe risks and can be fatal if untreated. These infections result in persistent microbial dissemination into the bloodstream and include conditions like endocarditis, thrombophlebitis, and endarteritis - primarily bacterial in origin. A specialized subset, catheter-related bloodstream infections (CRBSI), emerges when invasive devices, such as intravenous catheters, become colonized by microorganisms [3].

The escalating prevalence of multidrug-resistant (MDR) pathogens is a pressing concern in both nosocomial and community-acquired infections [4]. In 2017, the WHO identified 12 bacterial families in its antibiotic-resistant "priority pathogens" list, classified into CRITICAL, HIGH, and MEDIUM priority groups based on the urgency for novel antibiotics [5]. The critical category includes MDR bacteria like Acinetobacter, Pseudomonas, and Enterobacteriaceae, particularly threatening in hospitals and among patients requiring devices like ventilators or catheters. These pathogens exhibit resistance to vital antibiotics, including carbapenems and third-generation cephalosporins [5,6].

Rapid molecular techniques, leveraging polymerase chain reaction (PCR) principles, are widely utilized for identifying carbapenemase-producing genes. Available rapid molecular tests can detect the five prevalent carbapenemases (KPC, IMP, VIM, NDM, and OXA-48-like variants) within 24 hours, demonstrating sensitivities of 80-100%. Early identification of carbapenemase types is essential for precise targeted therapies and improved clinical outcomes [7]. Given the paucity of molecular data on resistance profiles of bacterial BSIs in India, particularly in intravascular infections, this study aims to investigate the phenotypic mechanisms of drug resistance and associated resistance genes in priority pathogens. The findings aim to enhance antibiotic stewardship and develop robust infection control strategies.

## Materials and methods

This study was structured as a cross-sectional investigation and commenced after obtaining ethical approval from the institutional ethics committee (EC/NEW/INST/2021/1618). All procedures adhered to established ethical standards and protocols. This research was conducted at the Institute of Microbiology, Rajiv Gandhi Government General Hospital (RGGGH), Madras Medical College (MMC), Chennai, Tamil Nadu, India. The study spanned seven months from September 1, 2023, to March 31, 2024, enrolling a total of 101 participants. The study encompassed patients admitted to cardiology, intensive care, and nephrology wards presenting with suspected BSIs. Individuals demonstrate clinical manifestations and echocardiographic evidence suggestive of infective endocarditis (IE), as defined by the modified Duke's criteria [8]. Additionally, patients with central lines in place for over 48 hours who exhibited clinical indicators of CRBSI, such as fever, chills, rigors, hypotension, or localized catheter site infections, were included.

Eligibility criteria comprised patients aged 18 years and above with intravascular infections, including IE, CRBSI, mycotic aneurysms, and suppurative thrombophlebitis. Exclusion criteria encompassed individuals younger than 18 years, as well as those with suspected extravascular BSIs resulting from conditions such as urinary tract infections, pneumonia, intra-abdominal infections, or skin and soft tissue infections. Patients with central venous catheters (CVCs) inserted before hospital admission were similarly excluded.

All specimen collections were performed after obtaining informed consent. Comprehensive demographic and clinical data were documented, encompassing patient history, clinical presentation, fever duration, catheterization details, echocardiographic findings, and laboratory results. For cases of suspected CRBSI, paired blood samples (10 mL each) were collected simultaneously from the catheter lumen and peripheral vein under rigorous aseptic conditions. The distal 5 cm of the catheter tip was aseptically removed, transported to the laboratory within 30 minutes, and processed using the Maki semi-quantitative method.

In patients suspected of IE, three sets of blood cultures were obtained within one hour, before the initiation of empirical antimicrobial therapy. If initial cultures yielded negative results after 24 hours, two additional sets were collected to detect persistent bacteremia. All blood cultures were processed using the automated BACT/ALERT system. Positive cultures were subjected to Gram staining, subculturing on MacConkey and blood agar media, and further species identification through biochemical characterization. The semi-quantitative Maki method was employed to process catheter tips, involving rolling the segment on sheep blood agar to distinguish infection from contamination.

Phenotypic resistance mechanisms, including extended-spectrum beta-lactamase (ESBL) production, carbapenemase activity (assessed through CARBA NP testing), and methicillin resistance, were evaluated using standardized protocols. Antimicrobial susceptibility testing was performed using the Kirby-Bauer disk diffusion method and the determination of minimum inhibitory concentrations for select antimicrobials. Molecular characterization was conducted to identify antibiotic resistance genes, including blaKPC, blaNDM, blaCTX-M, blaTEM, OXA23, OXA48, and mecA, using PCR. Observed resistance patterns were correlated with the clinical profiles of WHO priority pathogens.

Data were analyzed using IBM SPSS Statistics Version 31.0 (IBM Corp., Armonk, NY) [9]. Means and proportions were reported with 95% confidence intervals (CIs). A scatter plot diagram was employed to correlate microbial growth with catheter duration (in days), enabling visual assessment of infection trends over time.

## Results

Patient demographics and clinical characteristics

Age Distribution

Of the 101 patients, the majority belonged to the 40-60 year age group (55.45%), with the 50-60 year subgroup alone comprising 36.6%. This age range is often more immunocompromised due to advancing age, contributing to elevated exposure risks. Correspondingly, bloodstream infections were notably more frequent in this cohort, as illustrated in Table 1.

**Table 1:** Age-Wise Distribution of Patients With Bloodstream Infections (n = 101)

S. No	Age Group, Years	Frequency (%)
1	Below 30	25 (24.75%)
2	40–60	56 (55.45%)
3	Above 60	20 (19.80%)
4	Total	101 (100.00%)

Gender Distribution

Of the total patients, 71 cases (70%) were male, whereas 30 cases (30%) were female. This male predominance highlights a potential gender-based variation in susceptibility or healthcare-seeking behavior, and the peak age of occurrence of the disease was between 50 and 61 years.

Ward Distribution

Of the total study population (n = 101), 75% were admitted to the nephrology ward, 15% to the cardiology ward, and 10% to the ICU

Clinical diagnosis

The most common diagnosis was CRBSI, observed in 86 cases (85%), and followed by IE in 15 cases (15%).

Duration of Fever

The fever duration varied significantly among patients, as shown in Table 2. The longest fever duration recorded was 40 days in a patient diagnosed with infective endocarditis.

**Table 2:** Patient Stratification by Fever Duration

S. No	Fever Duration, Days	Frequency (%)
1	0–7	61 (60.40%)
2	8–15	25 (24.75%)
3	16–40	15 (14.85%)
4	Total	101 (100.00%)

Central venous catheter (CVC) characteristics

Site of CVC Insertion: All patients had internal jugular vein (IJV) catheterization (100%, n = 101).

Indication for CVC Placement

Of the total study population requiring CVC placement, the predominant indication was for hemodialysis. A majority (68%, n = 69) were undergoing treatment for chronic kidney disease (CKD), followed by 12% (n = 12) for acute kidney injury (AKI). The remaining 20% (n = 20) required CVC placement due to other critical conditions such as sepsis and multi-organ dysfunction. This distribution highlights the significant burden of renal disorders in patients with intravascular infections.

Duration of Catheter Placement

Of the total 101 catheterized participants, 49 (48.5%) exhibited microbial growth, while 52 (51.5%) showed no growth. This near-equal distribution underscores the importance of stratifying risk based on catheter duration. Notably, microbial growth increased progressively with longer indwelling periods. The data suggest a strong time-dependent association between catheter duration and colonization risk, reinforcing the need for timely catheter removal and stringent infection control protocols, as depicted in Table 3.

**Table 3:** Distribution of Microbial Growth by Catheter Duration in Culture-Positive Cases (n = 49) SD: standard deviation

S. No	Catheter Duration, Days	Mean ± SD, Days	No. of Growth (%)
1	1-10	5 ± 3	1 (2.04%)
2	11-20	15 ± 3	8 (16.33%)
3	21-30	25 ± 3	11 (22.45%)
4	31-40	35 ± 3	13 (26.53%)
5	41-50	45 ± 5	16 (32.65%)
Total			49 (100.00%)

Laboratory findings

Total Leukocyte Count (TLC)

The TLC varied among patients, ranging from 7,000 to 28,000 cells/mm³, with an average count of 14,200 cells/mm³, as shown in Table 4.

**Table 4:** Prevalence of Mild and Severe Leukocytosis in the Study Population

S. No	Leukocyte Range, cells/mm³	Category	Count (%)
1	4,000–11,000	Normal	12 (11.88%)
2	11,001–15,000	Mild leukocytosis	38 (37.62%)
3	>15,000	Severe leukocytosis	51 (50.50%)
4	Total		101 (100.00%)

Culture Reports

Blood cultures revealed positive growth in 48.5% of cases (n = 49), while 51.5% (n = 52) showed no growth.

Microbial Distribution

The distribution of organisms in the cohort is depicted in Table 5. Table 6 summarizes the prevalence of key antimicrobial resistance genes among Gram-negative and Gram-positive bacterial species isolated from BSIs

**Table 5:** Microbial Profile of Bloodstream Infections

Organisms	Count (%)
No growth	52 (51.5%)
Klebsiella pneumoniae	14 (13.9%)
Pseudomonas species	10 (9.9%)
Staphylococcus aureus	8 (7.9%)
Enterococcus species	4 (4%)
Streptococcus viridans	2 (2%)
Acinetobacter species	6 (5.9%)
Escherichia coli	5 (5%)

**Table 6:** Prevalence of Key Antimicrobial Resistance Genes Among Gram-Negative and Gram-Positive Bacterial Species Isolated from Bloodstream Infections

Resistance Gene	Klebsiella pneumoniae (n = 14)	Pseudomonas species (n = 10)	Acinetobacter species (n = 6)	Escherichia coli (n = 5)	Staphylococcus aureus (n = 8)	Enterococcus species (n = 4)	Streptococcus viridans
ESBL (extended-spectrum beta-lactamase)	71.4% (10 cases)	10% (1 case)	50% (3 cases)	80% (4 cases)	NA	NA	NA
blaKPC (Klebsiella pneumoniae carbapenemase)	35.7% (5 cases)	-	50% (3 cases)	20% (1 case)	NA	NA	NA
blaNDM (New Delhi Metallo-beta-lactamase)	35.7% (5 cases)	-	66.7% (4 cases)	20% (1 case)	NA	NA	NA
blaCTX-M	71.4% (10 cases)	10% (1 case)	50% (3 cases)	80% (4 cases)	NA	NA	NA
blaTEM	28.6% (4 cases)	10% (1 case)	50% (3 cases)	60% (3 cases)	NA	NA	NA
OXA-23	-	-	83.3% (5 cases)	-	NA	NA	NA
OXA-48	35.7% (5 cases)	-	50% (3 cases)	20% (1 case)	NA	NA	NA
MecA Gene (Methicillin Resistance)	NA	NA	NA	NA	62.5% (5 cases)	NA	NA

Antibiotic susceptibility patterns (AST) with resistance markers

**Figure 1:**
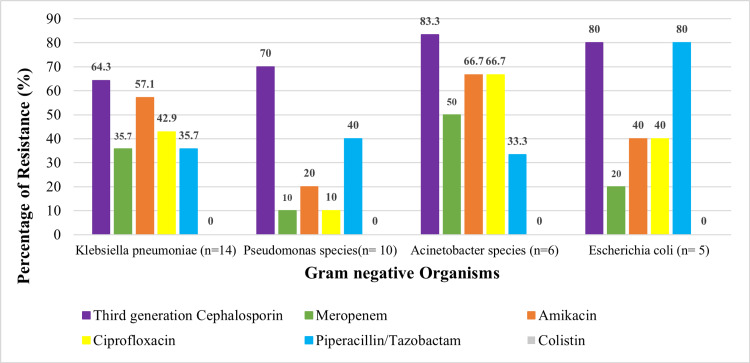
Antimicrobial Resistance Patterns Among Bloodstream Pathogens - Gram-Negative Organisms

**Figure 2:**
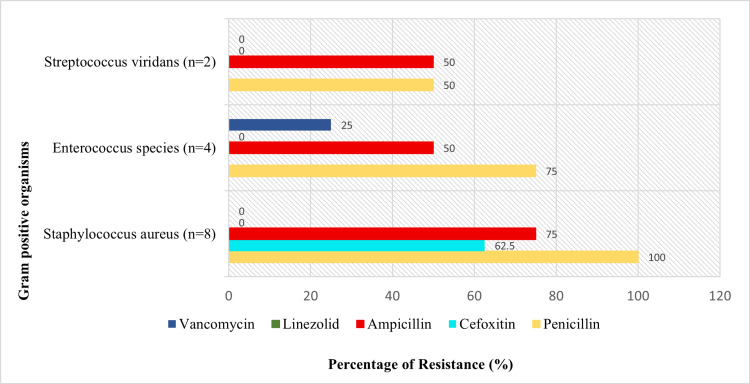
Antimicrobial Resistance Patterns Among Bloodstream Pathogens - Gram-Positive Organisms

Figures 1-2 illustrate the antibiotic susceptibility patterns (AST) with resistance markers.

Clinical outcomes

Among the 101 patients studied, targeted antibiotic therapy was effective in 70% of cases (n = 70), demonstrating clinical improvement without the need for additional intervention. Conversely, 30% of patients (n = 31) required escalation to broad-spectrum antibiotics, indicating initial treatment limitations and the presence of more resistant or complicated infections.

Complications

Clinical complications observed in the study cohort included persistent bacteremia in 7.9% of patients (n = 6), septic shock in 3.9% (n = 4), and multi-organ dysfunction in 1.9% (n = 2).

## Discussion

CRBSI and IE represent formidable challenges in hospitalized patients, particularly those undergoing CVC. The study identified Klebsiella pneumoniae as the predominant pathogen, followed by Pseudomonas species and Staphylococcus aureus. This aligns with previously documented epidemiological trends, reinforcing that Gram-negative bacteria, especially Klebsiella pneumoniae, constitute the leading etiological agents in nosocomial bloodstream infections [10,11]. Notably, resistance rates among Klebsiella pneumoniae isolates exhibited alarming levels, particularly against third-generation cephalosporins and carbapenems, underscoring the escalating crisis of antimicrobial resistance in hospital settings. Evidence suggests that infections caused by carbapenem-resistant Klebsiella pneumoniae (CRKP) are associated with heightened mortality rates and extended hospital stays, complicating clinical management and therapeutic outcomes [12,13].

Antimicrobial resistance in Klebsiella pneumoniae has garnered global attention, particularly following the emergence of hypervirulent strains harboring carbapenemase genes. The Global Antimicrobial Resistance and Surveillance System (GLASS-EAR) has reported a surge in hypervirulent Klebsiella pneumoniae (hvKp), notably the sequence type ST23, across multiple WHO regions. These strains exhibit heightened virulence and adaptability, rendering both immunocompetent and immunocompromised individuals susceptible to severe infections [14]. Given their rapid proliferation, reinforcing molecular diagnostics and surveillance remains critical for early detection and containment.

The resistance mechanisms of Klebsiella pneumoniae predominantly involve ESBL production and carbapenemase expression, leading to marked resistance against cephalosporins and carbapenems. The widespread dissemination of CRKP has drastically restricted available treatment regimens. Nevertheless, studies indicate that colistin and tigecycline remain viable last-resort therapeutic options. Furthermore, combination therapy involving colistin and tigecycline has demonstrated enhanced efficacy against colistin-resistant strains, presenting a potential strategy to counteract treatment failures [15].

A comprehensive investigation conducted in a tertiary hospital in Bucharest, Romania, documented a stark increase in antimicrobial resistance among Klebsiella pneumoniae isolates between 2019 and 2021. Resistance rates against third-generation cephalosporins, carbapenems, aminoglycosides, and fluoroquinolones have shown a steep upward trend, reinforcing concerns about the persistence of MDR strains. Intriguingly, a decline in resistance to trimethoprim/sulfamethoxazole (TMP/SMX) was observed, suggesting its potential reconsideration as a therapeutic option in CRKP infections [16].

Half of the study population exhibited profound leukocytosis (>15,000 cells/mm³), a well-recognized marker of systemic inflammation and infection severity. Elevated leukocyte counts have been consistently associated with increased morbidity and mortality in bloodstream infections. The correlation between leukocytosis and sepsis progression highlights its significance in prognostic evaluation and therapeutic intervention strategies [17,18]. All patients in the study had undergone IJV catheterization, with 68% requiring CVC placement due to CKD. Prolonged catheter placement has been implicated in biofilm formation, which subsequently predisposes patients to BSIs. The study found an average catheter duration of 14 days, which aligns with recommended guidelines advocating for timely removal to mitigate infection risks [19,20].

A noteworthy finding was the detection of ESBL in 11.4% of cases, along with carbapenemase genes (blaKPC, blaNDM, blaCTX-M, and blaTEM). The presence of OXA-type carbapenemases (OXA-23, OXA-48) further compounds therapeutic challenges, necessitating the exploration of combination therapies and novel antimicrobial agents. This highlights the evolving complexity of antimicrobial resistance and the imperative for precision-targeted therapeutic strategies [21,22]. Despite evidence-based antimicrobial therapy, 5% of patients required escalation to broad-spectrum antibiotics, underscoring the gravity of resistant infections and the ongoing struggle against therapeutic inadequacy. Delayed diagnosis and suboptimal empirical treatment were identified as contributing factors to poor clinical outcomes. Literature suggests that BSIs caused by ESBL-producing Enterobacteriaceae (ESBL-PE) correlate with elevated mortality risks, particularly in immunocompromised individuals [23,24].

Limitations

This study has a few limitations, notably its single-center design and short duration, which may affect the broader applicability of its findings. Additionally, the molecular analysis focused on a select group of resistance genes, potentially missing other relevant mechanisms. 

## Conclusions

This study reveals a high burden of CRBSIs and IE, especially in patients with renal comorbidities undergoing CVC. The frequent detection of ESBL producers and carbapenemase genes - blaNDM, blaKPC, and OXA variants - signals an alarming rise in MDR intravascular pathogens. These findings advocate for the integration of molecular diagnostics into routine workflows to enable rapid resistance profiling. Strengthened antimicrobial stewardship, strict infection control, and optimized catheter protocols are essential to curb infection rates. Notably, prolonged catheter dwell time correlates with microbial colonization, underscoring the need for timely removal. Future research should prioritize targeted therapies, novel antimicrobials, and real-time molecular surveillance to improve outcomes and contain resistance spread.
